# Antioxidants in Greek Virgin Olive Oils

**DOI:** 10.3390/antiox3020387

**Published:** 2014-05-13

**Authors:** Nick Kalogeropoulos, Maria Z. Tsimidou

**Affiliations:** 1 Laboratory of Chemistry, Biochemistry, Physical Chemistry of Foods, Department of Nutrition and Dietetics, School of Health Science and Education, Harokopio University, 70 El. Venizelou Str., Athens 17661, Greece; 2 Laboratory of Food Chemistry and Technology, School of Chemistry, Aristotle University of Thessaloniki, Thessaloniki 54124, Greece; E-Mail: tsimidou@chem.auth.gr

**Keywords:** Greek virgin olive oil, polyphenols, tocopherols, squalene, triterpenic acids

## Abstract

Greece is ranked third after Spain and Italy in virgin olive oil production. The number of Greek olive cultivars—excluding clonal selections—is greater than 40; however, more than 90% of the acreage is cultivated with 20 cultivars, adapted to a wide range of environmental conditions. Greek virgin olive oils, produced mainly with traditional, non-intensive cultivation practices, are mostly of exceptional quality. The benefits of consuming virgin olive oil, originally attributed to its high oleic acid content, are now considered to be the combined result of several nutrient and non-nutrient phytochemicals. The present work summarizes available data regarding natural antioxidants in Greek virgin olive oils (VOO) namely, polar phenolic compounds, tocopherols, squalene, and triterpenic acids. The literature survey indicated gaps in information, which should be filled in the near future so that the intrinsic properties of this major agricultural product of Greece will be substantiated on a solid scientific basis.

## 1. Introduction

Greece is among the leading olive producing countries of the world, and is ranked third after Spain and Italy. The annual production is around 310,000 t olive oil produced from 531,000 oil farms, covering a total of ~730,000 ha, and the annual olive oil consumption is estimated around 220,000 t. [1]. Consumption models differ in the European Union (EU) three main producing countries, which account for around 80% of EU consumption: in Italy and Greece, the majority of oil consumed is extra virgin, whereas in Spain this category represents less than half of consumption. However, the general trend is towards the consumption of extra virgin oils. The number of Greek cultivars—excluding clonal selections—is greater than 40 and more than 90% of the acreage is cultivated with 20 cultivars, adapted to a wide range of environmental conditions [2]. These are: Agouromanakolia, Adramitini, Amigdalolia, Asprolia, Valanolia, Vasilikada, Gaidurelia, Dafnelia, Thiaki, Kalamon, Kalokerida, Karolia, Karidolia, Kothreiki, Kolimpada, Konservolia, Koroneiki, Koutsourelia, Lianolia Kerkiras, Mastoeidis (referred also as Athinolia or Tsounati), Mavrelia, Megaritiki, Mittolia, Strogilolia, Throumbolia, and Tragolia. The benefits of consuming olive oil, known since antiquity, were, at first, attributed to its high oleic acid content. However, it is now well established that these effects may also be due to the concerted action of several nutrient and non-nutrient phytochemicals which exert anti-oxidant, anti-inflammatory, anti-microbial and other activities [3]. Among them the polar phenolic compounds attracted the interest of researchers coming from different fields and countries worldwide. The goal of this review is to summarize available data on natural antioxidants reported in Greek VOO, namely polar phenolic compounds, tocopherols, squalene, and triterpenic acids. In this respect, factors affecting their qualitative composition and quantitative levels will be addressed when available data exist. The above mentioned compounds are found in the two edible commercial types of the product, namely the “extra virgin olive oil” (EVOO), and the “virgin olive oil” (VOO). Both products are produced by only physical and mechanical means and are differentiated in legislation in terms of limits in certain quality criteria (free acidity as % oleic acid, peroxide value, UV absorbance K_232_ and K_270_ values, organoleptic score) [4]. For the purpose of this review the term VOO is adopted not as a commercial category name, but to describe the product that is mechanically or physically extracted from olive fruits and treated till consumption by only mechanical and physical processes.

## 2. Polar Phenolic Compounds

### 2.1. Composition

The methanolic or aqueous methanol extract of VOO contains the polar phenolic compounds, which determine to a great extent the quality of the oil by improving its oxidative stability and contributing to its sensory properties, while, in addition, they are considered beneficial for the prevention of certain chronic diseases [5,6]. The good correlation between oxidative stability and the phenolic content of the oil has been established since the early 1970s [7]. The following decades the polar phenols content was shown to be the most important determinant of VOO stability [8,9,10,11] and the study of factors that control it became of major concern. The major classes of VOO phenolic compounds are flavonoids, lignans, simple phenols and secoiridoids, with the last two groups predominating. Individual phenolic compounds normally present in VOO are the following [12,13]: (i) benzoic acids and derivatives: 3-hydroxybenzoic acid, *p*-hydroxybenzoic acid, 3,4-dihydroxybenzoic acid, gentisic acid, vanillic acid, gallic acid, syringic acid; (ii) cinnamic acids and derivatives: *o*-coumaric acid, *p*-coumaric acid, caffeic acid, ferulic acid; sinapic acid; (iii) phenyl ethyl alcohols: tyrosol [(*p*-hydroxyphenyl)ethanol], hydroxytyrosol ((3,4-dihydroxyphenyl)ethanol); (iv) other phenol acids and derivatives: *p*-hydroxyphenylacetic acid, 3,4-dihydroxyphenylacetic acid, 4-hydroxy-3-methoxyphenylacetic acid, 3-(3,4-dihydroxyphenyl)propanoic acid; (v) dialdehydic forms of secoiridoids: decarboxymethyloleuropein aglycon (oleacin), decarboxymethyl ligstroside aglycon (oleocanthal); (vi) secoiridoid aglycons: oleuropein aglycon, ligstroside aglycon, aldehydic form of oleuropein aglycon, aldehydic form of ligstroside aglycon; (vii) flavonoids: (+)-taxifolin, apigenin, luteolin; (viii) lignans: (+)-pinoresinol, (+)-1-acetoxypinoresinol, (+)-1-hydroxypinoresinol; (ix) Other categories like the hydroxyisochromans 1-phenyl-6,7-dihydroxyisochroman, 1-(3-methoxy-4-hydroxy)phenyl-6,7-dihydroxy-isochroman are also mentioned in certain publications.

### 2.2. Health Benefits and Bioavailability

The health benefits of phenolic compounds of VOO are mainly due to the presence of the unique group of secoiridoids present in all parts of olive tree [5,6,14]. The most strongly substantiated health benefit seems to be their antioxidant activity and the protection these phenolic compounds exert on blood lipid oxidation. Antiinflammatory activity, anticarcinogenic potential, modulation of gene expression towards a protective mode for proteins participating in the cellular mechanisms involved in oxidative stress resistance, inflammation or lipid metabolism, and a numerous other health benefits [15,16,17,18,19,20,21,22] deserve further studies. The same applies to those on the bioavailability of individual compounds (hydroxytyrosol, tyrosol, oleuropein and aglycons, elenolic acid) or phenolic extracts rich in them.

### 2.3. Total Polar Phenolic Content of Greek VOO

Total polar phenolic content (TPC) is usually estimated by a colorimetric method based on the use of Folin-Ciocalteu reagent [8] and the results are expressed as mg of caffeic acid equivalents (CAE) or gallic acid equivalents (GAE) per kg of oil, depending on the standard used. It must be noted that no consensus is reached so far on the choice of standard. Irrespectively of the accuracy of the procedure [11] the TPC has been repeatedly proved to be a marker for VOO stability and is also related to taste characteristics It shows a great variability, reported to range from 50 to 1000 mg CAE/kg in VOO from Greece, Israel, Italy, Spain, and Turkey, with usual values between 100 and 300 mg CAE/kg, e.g., [5,23]. Higher levels are not always positively perceived by the consumers. Regarding taste perception, Italian VOOs had been categorized according to their TPC as “low” with 50–200, “medium” with 200–500 and “high” with 500–1000 mg GAE/kg [24,25]. The cultivar, climate and other environmental factors, harvesting time, the extraction process, and the conditions of packing, distribution, and storage are critical factors affecting the final phenolic content of VOO [24]. Among them cultivar and extraction technology are considered to predominate in determining the magnitude of TPC of olive oils. Boskou and his collaborators initiated the studies on the phenolic compounds of Greek virgin olive oil in late eighties [5]. Focusing on stability issues it was evidenced that the levels of TPC of VOO from different areas in Greece differ to a great extent (18.7–242.5, *n* = 24) [9]. The authors postulated then the importance of a group of unknown phenolic compounds for the overall VOO stability. The identity of those compounds has been nowadays fully clarified and will be discussed in a subsequent section.

The TPC content of Greek VOO has been repeatedly reported [9,11,26,27,28,29,30,31,32,33,34,35,36]. The results of a recent comprehensive study [28] which covered 5 harvesting years and involved 221 randomly collected EVOO samples, are presented in Table 1. The EVOO samples were of the Koroneiki, Tsounati and Adramytini cultivars and originated from the major Greek olive producing areas—Crete and Peloponnisos, and from two islands—Zakynthos in the Ionian Sea and Lesvos in E Aegean Sea. All the samples were prepared and analyzed for individual phenolics by ^1^H and ^31^P-NMR and the results presented are the sum of individual phenolics. Given that sample preparation and analysis were conducted by the same laboratory employing the same analytical techniques, the data of Table 1 clearly indicate that cultivar and geographical origin are not the only factors determining the levels of TPC in EVOO.

**Table 1:** TPC reported for Greek virgin olive oils, determined by ^1^H and ^31^P-NMR [28].

Origin	Cultivar	Total Phenol Content (Range) (mg/kg)
**2007–2008**		
Crete (Chania)	Koroneiki (*n* = 26)	138–441
	Tsounati * (*n* = 9)	173–641
Crete (Rethimnon)	Koroneiki (*n* = 25)	65–320
	Throubolia (*n* = 5)	118–294
Crete (Sitia)	Koroneiki (*n* = 23)	95–351
**2005–2006**		
Crete (Heraklion)	Koroneiki (*n* = 2)	53–92
Crete (Chania)	Koroneiki (*n* = 6)	25–120
Peloponnisos (Lakonia)	Koroneiki (*n* = 1)	95
Peloponnisos (Lakonia	Tsounati (*n* = 7)	72–208
Peloponnisos (Messinia)	Koroneiki (*n* = 13)	37–118
Zakynthos	Koroneiki (*n* = 12)	49–142
Lesvos	Adramytini (*n* = 5)	31–163
**2004–2005**		
Crete (Heraklion)	Koroneiki (*n* = 2)	130–205
Crete (Rethimnon)	Koroneiki (*n* = 1)	83
Crete (Chania)	Tsounati (*n* = 5)	91–216
Peloponissos (Lakonia)	Koroneiki (*n* = 5)	87–225
Peloponissos (Messinia)	Koroneiki (*n* = 16)	39–189
Zakynthos	Koroneiki (*n* = 9)	67–145
Lesvos	Adramytini (*n* = 8)	23–212
**2003–2004**		
Crete (Chania)	Koroneiki (*n* = 2)	145–248
Peloponissos (Lakonia)	Koroneiki (*n* = 3)	40–183
Peloponissos (Lakonia	Tsounati (*n* = 9)	72–225
Peloponissos (Messinia)	Koroneiki (*n* = 9)	82–175
**2002–2003**		
Crete (Heraklion)	Koroneiki (*n* = 7)	77–164
Crete (Heraklion)	Tsounati (*n* = 2)	46–86
Crete (Chania)	Koroneiki (*n* = 2)	67–108
Crete (Chania	Tsounati (*n* = 2)	89–114
Peloponissos (Lakonia)	Tsounati (*n* = 5)	85–134

*: “Tsounati” cultivar is also referred as “Mastoeidis” and “Athinolia” [2].

The data so far indicate that Koroneiki cultivar may give VOOs with TPC higher than 250 mg/kg. What is not clear from the experimental design of the publication is the reason for the discrepancies observed as sampling was rather at random. The potential of a cultivar regarding TPC is not always reflected to the products that reach consumers. For example, in olive oil producing countries till recently significant amounts of olive oil were marketed in bulk. In an attempt to characterize the quality of these oils, a study undertaken on 120 olive oil samples purchased in bulk and sampled from Greek households all over the country, revealed that only 17 samples belonged to the EVOO category, 22 belonged to the VOO category, while 81 samples were categorized as non edible, because the value of at least one analytical parameter related to quality deviated from that required for EVOO or VOO [36]. TPC values for EVOO samples ranged from 65 to 218 mg CAE/kg (mean 117 mg /kg) being lower than the respective of EVOO obtained directly from olive mills (range 32–339 mg CAE/kg, mean 130 mg/kg with 40% of samples containing >150 mg/kg) [11]. The TPC of VOO samples purchased in bulk were—as expected—lower than the EVOO ones, ranging between 22 and 187 mg CAE/kg (mean value 82 mg/kg), with the 82% of the samples containing <100 mg/kg [36].

### 2.4. Individual Phenolic Compounds in Greek VOO

Typical levels of individual phenols are difficult to establish due to natural variability and strong dependence on oil age and post-production history. Normally, fresh oils contain more complex forms of secoiridoid aglycons, whereas in stored oils free phenols predominate [37]. The qualitative and quantitative data for individual phenols are additionally influenced by the extraction procedures and the analytical techniques followed. The more polar part of the methanol-water extract contains free phenols and phenolic acids, while the less polar part contains aglycones of oleuropein and ligstroside (the hydroxytyrosol and tyrosol esters of elenolic acid), deacetoxy and dialdehydic forms of these aglycones, the flavones luteolin and apigenin, the lignans 1-acetoxypinoresinol and pinoresinol, and also elenolic acid and cinnamic acid [37]. For the identification-quantitation of individual phenols in the polar fraction of Greek olive oils, gas chromatography coupled with mass spectrometry, high performance liquid chromatography with electrochemical, UV, diode array or fluorescence detectors, as well as ^31^P and ^1^H-NMR spectroscopy have been applied [13]. The phenolic compounds reported so far in Greek VOO, presented in Table 2, are among those normally reported in olive oil [12]. Their concentrations show wide variability attributable to differences in cultivars, pedoclimatic conditions, harvesting time, technological manipulations, the extraction protocols and analytical techniques applied for their isolation and determination. Besides the phenolic species prevailing in VOO, namely hydroxytyrosol, tyrosol and their derivatives. The presence of low concentrations of lignans, phenolic acids and flavonoids has been also reported. In addition, the presence of an ester of tyrosol with a dicarboxylic acid was reported in EVOO from Crete and Peloponnisos after fractionation with solid-phase extraction and analysis by RP-HPLC [38]. Coupling HPLC with postcolumn solid-phase extraction to NMR Spectroscopy (LC-SPE-NMR) for the direct analysis of polar fraction of EVOO from Crete and Lesvos Island, enabled the identification and structure elucidation of simple phenols (hydroxytyrosol, tyrosol, vanillic acid, vanillin, *p*-coumaric acid, hydroxytyrosol, and tyrosol acetates), lignans (pinoresinol and 1-acetoxypinoresinol), flavonoids (apigenin and luteolin), and a large number of secoiridoid derivatives, among them elenolic acid (not a phenol) and the dialdehydic form of elenolic acid lacking a carboxymethyl group which was detected for the first time in olive oils [39].

The same authors reported for the first time the spectroscopic information for ligstroside aglycon. Recently the direct measurement of oleocanthal (dialdehydic form of decarboxymethyl oleuropein) and oleacein (dialdehydic form of decarboxymethyl ligstroside) levels in olive oil by quantitative ^1^H-NMR was applied to the study of 175 monovarietal commercial Greek and Californian EVOO samples [40]. It was found that the concentrations of these health promoting compounds [41] varied significantly—from non detectable to 355 mg/kg—being higher at the early ripening stages. It was also reported that there are olive varieties that produce oil with low content of both compounds, independently of geographic origin and harvest time, in line with observations made by Servili and Montedoro (2002) [42]. Recently, Agiomyrgianaki *et al.* (2012) [28] reported the results of a study on 221 monovarietal EVOO samples obtained during 5 harvesting years from the main Greek olive oil producing areas—Crete and Peloponnisos—as well as from two Greek islands. The authors determined total and free hydroxytyrosol and tyrosol, together with syringaresinol, acetoxypinoresinol, *p*-coumaric acid, homovanillyl alcohol, luteolin, apigenin and pinoresinol by employing NMR. The levels of the prevailing phenols in the oils of this study are summarized in Table 3. The concentrations of the other compounds were rather low and are not presented. From the data of Table 3 it appears that Koroneiki EVOO from Crete was richer in total and free hydroxytyrosol compared to the respective EVOO from Peloponnisos and Zakynthos. The same trend was observed for total hydroxytyrosol in Mastoeidis EVOO from Crete compared to the respective Mastoeidis oils from Peloponnisos. No similar trend was observed for the other phenolic species presented in Table 3.

### 2.5. Factors Affecting Polar Phenol Composition and Levels

The polar phenolic content in olive oils varies in quantity and quality, being closely related to the cultivar, agricultural techniques used in cultivation, soil composition, climate, the ripening stage of olive drupes at harvest, and the processing technique and storage.

#### 2.5.1. Olive Fruits Maturation

During maturation the phenolic profile of olive fruits is significantly modified due to enzymic activity in a manner closely related to cultivar characteristics [43]. The oleuropein content is constantly reduced and is at a minimum in overripe drupes. At the same time, demethyl-oleuropein replaces oleuropein in about the same amounts [44]. The fruit of *O. europea* accumulates only glycosylated derivatives of oleuropein, which are probably less toxic than aglycones. It is difficult to define the optimal stage of maturity in order to determine harvesting time. Delaying the harvest time may be crucial in maintaining oleuropein derivatives in olive oil—because of differences in their distribution between the oil and aqueous phases—and balancing bitter to pungent taste [45,46]. In a study of Tunisian oils, TPC increased progressively as olives matured and decreased in the final ripening stage [47]. This was not the case for EVOO from trees of the Koroneiki cv. grown in Greece, as a marked increase of TPC in parallel with fruits’ maturation was observed in oils from Crete Island [31] and Southern Peloponnisos [34,35]. On the contrary, TPC in oils of the Throumbolia cv. from Crete remained almost constant during olives ripening [31]. Regarding individual phenolics, ripening of Koroneiki fruits grown in Southern Peloponnisos [35] caused an increase in the EVOO content of free hydroxytyrosol, *p*-coumaric acid and *p*-hydroxybenzoic acid. In another study of Koroneiki EVOO from the same region [34] increased values of tyrosol, hydroxytyrosol and homovanillic alcohol were observed in the late stage of fruits’ maturation (January). In Koroneiki EVOO from Crete, ripening caused an increase of total tyrosol, total hydroxytyrosol, oleuropein aglycon, dialdehydic form of elenolic acid linked to hydroxytyrosol and a decrease of the dialdehydic form of elenolic acid linked to tyrosol [31], something that does not agree with the accumulation of phenols in olive drupes. In a recent publication for ripening of olives in hot climates, no consistent reduction of TPC in oils from Barnea, Coratina, and Picual cv cultivated in Jordan Valley, Israel [48].

#### 2.5.2. Cultivar and Geographical Origin

Olive polyphenols depend both on the cultivar and the origin area, without overlooking pedoclimatic conditions [49,50]. The influence of cultivar and geographical origin on TPC of Greek VOO is difficult to be documented with certainty, as most of the available data refer to Koroneiki cv and as it was shown in previous session (Paragraph 2.3 and Table 1) cultivar and geographical origin alone are not sufficient to explain the TPC content. Regarding individual phenolic compounds it is not so easy to reach to conclusions from literature data, due to differences in sample preparation and analysis and in lack of documentation for the history of samples.

#### 2.5.3. Organic *vs.* Conventional Cultivation

Data comparing the phenolic compounds of organic *vs.* conventional olive oils are scarce. In a comparative study conducted in Southern Peloponnisos, Koroneiki EVOO obtained from olive drupes of organic and of non-organic cultivation that were sampled at four successive ripening periods for two crop years, were analysed for several quality indices and phytochemicals content. Statistical analysis demonstrated that organic Koroneiki cv. olive oils exhibited higher TPC for the two crop years (317.2 and 306.6 mg CAE/kg) compared to the conventional ones (273.7 and 237.0 mg CAE/kg) [34] in accordance with the study by Ranalli and Contento (2010) [51]. Among the individual phenols determined free tyrosol and hydroxytytosol did not differ according to the cultivation method, the latter being in line with the findings of Ninfali *et al.* (2008) [52] who did not obtain consistent results regarding quality, phenolics and antioxidant capacity of olive oils extracted from organic or conventional Leccino and Frantoio olives in a three-year study, It seems probable that genotype and climate variations exert more marked effects than the cultivation method.

#### 2.5.4. Water Availability

Several reports have indicated that the amount of phenolic compounds in oils obtained from drought-stressed crops is usually higher compared to irrigate ones [53,54,55]. This was confirmed in Greek oils; reduced water supply led to elevated TPC compared to the control in VOO produced from olives of the Konservolia cv. in Thessaly, Central Greece [56]. The same trend was observed in EVOO produced during 2 successive crop years from Koroneiki olive trees grown in Crete. During both harvesting periods the TPC of EVOO obtained from non-irrigated trees (248.4 and 474.1 mg GAE/kg, respectively) was higher than that of the irrigated trees (201.5 and 403.6 mg GAE/kg, respectively) [29]. In addition, EVOO produced from the non-irrigated trees contained more total hydroxytyrosol, oleuropein aglycon, and dialdehydic form of elenolic acid linked to hydroxytyrosol, compared to EVOO from the irrigated trees. However, when olive trees are cultivated in areas with different pedoclimatic conditions than those in which they have been evolved and adapted, the phenolic content may follow different patterns. This was the case of cultivating the Spanich cultivar Arbequina in Tunisia under different irrigation conditions; it was found that phenolics in the respective olive oils were proportional to the amount of irrigation water [57], contrary to the results reported above.

#### 2.5.5. Milling, Malaxation and Extraction Technology

The delicious taste and aroma of good quality VOO are partially due to complex phenols with bitter taste and volatile compounds generated during the milling and malaxation steps of oil production mainly through the activity of endogenous enzymes which influence the relationship between the volatile and the phenolic components in VOO [58].

##### 2.5.5.1. Milling/Crushing

During crushing, the main glycosides present in the fruit are hydrolyzed by action of endogenous β-glucosidases leading to the formation of secoiridoid aglycons. Hammer crushers are preferred to stone crushers for the production of VOO with increased phenolic content [59]. In this case, the conditions of kneading (temperature, time) [60] and even the rotation rate [61] may be critical factors for the amount of total phenols, as an increase from 2200 rpm to 2900 rpm caused about 40% increase in the antioxidant power of the polar fraction due to a better fragmentation of olive tissues which in turn increased the rate of enzymic reactions [61].

The effect of olive crushing parameters was demonstrated in a study with Greek olives, where significantly higher TPC values for oils obtained using hammer crusher operating at 3000 and 2500 rpm were reported [62]. In the same study the authors compared screens with 5 or 6 mm hole gratings and noticed that oils obtained using the 6-mm screen size presented higher TPC values. We must note here that higher hammer crusher turns rate causes an increase of the crushed pastes’ temperature, and furthermore a higher peroxidase (POD) release, because of a deeper fragmentation of the seeds which contain high amounts of such enzymes [63]. Consequently, a hammer crusher may provide olive oil with lower polyphenols content compared to a blade crusher or to destoning before malaxation [64].

##### 2.5.5.2. Malaxation

Malaxation modifies the qualitative and quantitative composition of VOO phenolic compounds [24,25,50,58,65,66]. Glycosidases that are present in the olive fruit and consequently in the paste result in the formation of aglycone forms of secoiridoids, which are subsequently oxidized by the oxidizing enzymes of the polyphenoloxidase (PPO), peroxidase (POD) and lipoxygenase (LOX) pathways. These enzymes are triggered by the milling of olives, and are active during the malaxation step. Both classes of enzymes have oxygen as a co-factor, therefore the presence of oxygen in the headspace of the malaxation machine favor oxidation of phenols reducing their concentration in the paste and in the oil. For this reason the headspace composition and the time of exposure of olive paste to air during the malaxation is considered a processing parameter that can be used to control endogenous oxidoreductases such as PPO, POD and LOX [58]. The effect of temperature should not be overlooked, as a positive relationship between the temperature of malaxation and the phenolic concentration in olive pastes and in virgin olive oils has been observed in sealed malaxing machines and was attributed to differences in the thermal stability of PPO and POD [63]. The extent of compositional changes triggered by milling and malaxation was shown in a recent study on the fate of olive key metabolites during Koroneiki EVOO extraction in a 2-phase olive mill in Lakonia, Peloponnisos [67]. The authors employed an HPLC-Orbitrap platform and identified fifty-two components belonging to phenyl alcohols, secoiridoids, flavonoids, triterpenes, and lactones in drupes, paste, first oil and final oil. It was shown that only a small portion of the beneficiary metabolites ended up in the final product, as only twenty components were detected in the final oil. The cocentrations of hydroxytyrosol, tyrosol, oleacein, and oleocanthal increased significantly from olive drupes to paste but were minimized in the final oil, while oleuropein, ligstroside, aldehydic form of oleuropein aglycon, and aldehydic form of ligstroside aglycon decreased from drupes to paste and also minimized in the final oil.

##### 2.5.5.3. Extraction Technology

The extraction system also affects the TPC and the individual phenols profile of the final product. The comparison of three extraction systems (pressure, 2-phase, and 3-phase) for a period of 5 crop years, revealed that oils obtained by 2-phase decanters contained more total phenols and exerted superior oxidative stability, while presenting a slightly higher index of bitterness [64]. The superiority of 2-phase extraction towards the greater recovery of phenolic compounds has been repeatedly reported [60,68]. The lower phenol content of the oil extracted in 3-phase centrifuges is mainly due to the addition of water, which reduces the concentration of the polar phenolic compounds. This was also demonstrated in EVOO produced with 2-phase and 3-phase decanters from Coratina and Koroneiki olives grown in Pescara, Italy and Chania, Greece. EVOO produced with the 2-phase decanter exhibited higher TPC, total tyrosol, oleuropein aglycon, dialdehydic form of elenolic acid linked to hydroxytyrosol and dialdehydic form of elenolic acid linked to tyrosol levels, compared to EVOO produced with the 3-phase decanter [30,69]. The same trend was recently reported for TPC, hydroxytyrosol, tyrosol, vanillin and homovanillic alcohol levels in cold pressed EVOO produced with 2-phase and 3-phase decanters from olives of the Koroneiki cultivar collected from the same organic olive field in Messenia, Peloponnisos [70].

#### 2.5.6. Filtration

Filtration results in transparent brilliant olive oils with increased shelf life, as a result of moisture content reduction. Prior to bottling, most companies apply a filtration step to remove suspended solids and reduce VOO moisture content, aiming both to increase product’s shelf life and to improve its visual acceptability by consumers [71]. Literature data indicate that filtration reduces the oxidative stability as the removal of moisture affects mainly the polar fraction of EVOO, responsible of its oxidative stability [71,72]. Regarding the effect of filtration on individual phenolics, controversial results have been reported in the literature; total phenolic content and phenolic alcohols decrease, while secoiridoids increase [72], probably due to different extractability of different phenolic classes in the presence or absence of water droplets which alters the qualitative and quantitative results obtained from VOO analysis. In a study employing Greek EVOO from Crete, Athens, and Peloponnisos it was found that unfiltered (veil) oils from Crete contained 10%–19% more TPC compared to the respective filtered ones; unfiltered oils from Athens contained 22%–53% more TPC than the filtered ones, while filtration had practically no effect on TPC content of EVOO from Peloponnisos [26].

## 3. Tocopherols

Tocopherols and tocotrienols are lipophilic phenolic antioxidants appreciated for their vitamin E activity. They are found in natural oils and fats in their free form. Early interest in these natural antioxidants was related to their contribution to the stability of lipid substrates. Thus, emphasis was paid to the role of the most active members, *i.e.*, γ- and δ- tocopherols and their role in shelf life of unsaturated vegetable oils or model systems (e.g., methyl esters of linoleic acid). For many years the role of α-tocopherol, the most active vitamer, as an antioxidant was rather overlooked. It is established that antioxidant activity of tocopherols is concentration dependent, decreasing above an optimum level, which is different for each individual compound. Excess levels may lead to a prooxidant effect possibly due to a faster hydroperoxide decomposition and, consequently, to acceleration of the propagation kinetics [73]. Virgin olive oil (VOO) is characterized by the almost exclusive presence of α-tocopherol (α-T) that counts for the 90%–95% of the total tocopherol content so that its stability in the dark, under light exposure or upon thermal treatment is expected to be influenced by the presence of this tocol derivative. Past food chemistry reference books reported rather low levels of α-T for olive oils (<150 mg/kg). The concentration of α-T in a VOO depends on pedoclimatic, agronomic and technological factors. Genetic factors in some cases are becoming important. Current legislation on health claims gives an opportunity for advertisement of VOO as a good source of vitamin E and points to an urge for systematic knowledge of the potential of different olive cultivars and technological systems to yield extra VOO of high content in this bioactive compound. Moreover, its levels in commercial products should be also examined. Industrial and domestic handling conditions and practices are also critical factors for the maintenance of the prone to oxidation α-T. The recognition of the health benefits of polar phenolic compounds together with the fact that VOO is a good source of vitamin E are expected to influence consumption of VOO worldwide if their contents are in line with legislative requirements.

### Concentration of α-Tocopherol in Greek VOOs

The prevailing vitamer in VOO among the eight known ones in natural lipids is α-tocopherol. Natural α-tocopherol (α-T) presents the highest biopotency and its presence in the oil adds to the beneficial properties of the latter. Very recently, Tsimidou (2012) [74] reviewed the levels of α-tocopherol by producing country or zone worldwide and made clear that existing data do not cover all aspects for the dependence of individual tocopherols on genetic and other factors. As a general rule, it can be argued that γ-T presence is influenced by the genetic factor more than the other forms and that some cultivars contain rather high levels of it whereas others contain negligible ones. Ripeness and extraction system are not as critical as in the case of the polar phenolic compounds. Tsimidou (1985) [75] reported the first values for the α-tocopherol content of Greek virgin oils. Data were for oils from hydraulic and centrifugal systems used at that period in the major oil producing areas of Greece. Eighteen out of the 40 oils (harvest years 1981/1982 and 1982/1983) analysed by normal phase HPLC-fluorescence were obtained using the currently obsolete hydraulic pressure extraction system. Five of them having free acidity lower than 0.65% were found to contain a mean value of 134 mg/kg, whereas those of similar free acidities from the centrifugal systems presented even lower levels. VOOs of acidities higher than 2% (up to 5.26%) contained from 5.3 to 147 mg α-T /kg oil. The same author reported some interesting observations for the phenomenon of α-T regeneration upon refining process. The observations were confirmed for five sets of lampante-neutralised-bleached and deodorized oils (Elaiourgiki SYN.PE, Piraeus, Greece). The levels of α-T were found to increase from traces in lampante-neutralized and bleached oils to 3.5 mg/kg in the deodorized ones. Lower levels of tocopherols are expected in refined olive oils [76], but legislation does not allow consumption of them despite the fact that seed oils are mostly consumed refined. Almost 20 years after, Psomiadou *et al.* (2000) [77] published compositional data for a large number of Greek virgin olive oils obtained from different cultivars and regions all over Greece over three consecutive harvesting periods (1994/1995; 1995/1996; 1996/1997). The majority of oils had been obtained by three phase systems. The progress in the production of Greek virgin olive oils was obvious as >60% of the samples contained ~200 mg α-T/kg oil. The concentrations of β- and γ-tocopherols were varied from trace to 9 and 40 mg/kg, respectively. Oils from Koroneiki cultivar, were found to contain some of the highest values reported for world VOOs (*i.e.*, >300mg/kg). Extraction technology (hydraulic, two and three phase systems) was not found to be a critical factor contrary to what is known for the polar phenolic antioxidants of VOO, a finding perfectly in line with the lipophilic and hydrophilic nature of tocopherols and secoiridoids, respectively. The differences studied at three different periods of Koroneiki cultivar (Chania, Crete) collection were less than 30 mg/kg among the three systems [70]. Cultivar and agronomic practices seem to be more important factors. Stefanoudaki *et al.* (2009) [29] reported that irrigation may lead to a slight decrease in the total tocopherol content. Thus, they reported for Koroneiki cv. grown in Crete that, in two consecutive harvests, irrigation caused a decrease of ~90 mg/kg. The mean values for oils obtained when trees were regularly irrigated were ~200 mg/kg. Similar to above mean values were the values found for Greek commercial virgin olive oils (163–250 mg/kg, *n* = 25) analyzed on a date close to expiring one (max 18 months) [77]. Interesting was the finding that virgin olive oils purchased in bulk by consumers contained equally high levels of tocopherols despite the fact that the containers, distribution practice and consumer handling was not the recommended ones [36]. Studying correlations between stability of Greek VOO and the various types of antioxidants, Papadopoulos *et al.* (1993) [78] and Psomiadou *et al.* (2003) [79] supported that the contribution of α-T is lower than that of polar phenolic compounds but still important, especially as oil ageing progresses. Studies on model olive oil devoid of prooxidants and antioxidants proved the double character of α-T, *i.e.*, radical scavenger and radical propagator and its contribution in the resistance of oil at elevated hydroperoxide values [80].

**Table 2:** Individual phenolic compounds reported in Greek virgin olive oils.

Analytical Technique		Cultivar		*N*			Compounds			Reference	
HPLC		NS		24			Hydroxytyrosol, tyrosol			[9]	
HPLC various detectors		NS		Not provided			Hydroxytyrosol, tyrosol, vanillic acid, *p-*hydroxybenzoic acid, syringic acid, *o*-coumaric, *p*-coumaric acid, gallic acid, homovanillic acid, ferulic acid			[81]	
HPLC		NS Cloudy and filtered oils		6			Hydroxytyrosol, tyrosol			[26]	
HPLC		Koroneiki		8			Hydroxytyrosol, tyrosol, oleuropein aglycon, dialdehydic form of elenolic acid linked to OH-tyrosol, dialdehydic form of elenolic acid linked to tyrosol, tyrosol derivative, caffeic acid, vanillic acid			[27]	
LC-SPE-NMR		Koroneiki Kolovi		2 2			Hydroxytyrosol, tyrosol, hydroxytyrosol acetate, tyrosol acetate, a large number of secoiridoid derivatives including elenolic acid, vanillic acid, vanillin, *p*-coumaric acid, pinoresinol, 1-acetoxypinoresinol, apigenin, luteolin			[39]	
^31^P-NMR		Koroneiki Mastoeidis		2 2			Total and free hydroxytyrosol and tyrosol *, vanillin, vanillic acid, homovanillic acid, (+)-pinoresinol, (+)-1-acetoxypinoresinol, syringaresinol, luteolin, apigenin			[82]	
^1^H-NMR, ^31^P-NMR and HPLC		Koroneiki, Kolovi, Mastoeidis *		111			Total and free hydroxytyrosol and tyrosol, (+)-pinoresinol, (+) 1-acetoxypinoresinol, luteolin, apigenin			[83]	
^1^H-NMR, ^31^P-NMR		Koroneiki		131			Total and free hydroxytyrosol and tyrosol, *p*-coumaric acid, homovanillic alcohol, (+)-pinoresinol, (+)-1-acetoxypinoresinol, syringaresinol, luteolin, apigenin			[84]	
^1^H-NMR, ^31^P-NMR		Koroneiki		4			Total hydroxytyrosol and tyrosol, oleuropein aldehydic form, ligstroside aglycon, oleuropein and ligstroside aldehydic form, decarboxymethyl oleuropein and ligstroside dialdehydic form, *p*-coumaric acid, vanillin, vanillic acid, homovanillic alcohol, (+)-pinoresinol, (+)-1-acetoxypinoresinol, syringaresinol, apigenin			[85]	
^1^H-NMR, ^31^P-NMR		Adramitini, Koroneiki, Throumbolia, Mastoeidis		221			Total and free hydroxytyrosol and tyrosol, p-coumaric acid, homovanillic alcohol, (+)-pinoresinol, (+)-1-acetoxypinoresinol, syringaresinol, luteolin, apigenin			[28]	
HPLC		Koroneiki irrigated *vs.* not irrigated		6			Total hydroxytyrosol and tyrosol, p-coumaric acid, homovanillic alcohol, (+)-pinoresinol, (+)-1-acetoxypinoresinol, syringaresinol, luteolin, apigenin			[29]	
HPLC		Koroneiki 2- *vs*. 3-phase decanters		9			Total hydroxytyrosol and tyrosol, oleuropein aglycon, dialdehydic form of elenolic acid linked to OH-tyrosol, dialdehydic form of elenolic acid linked to tyrosol, tyrosol derivative			[30]	
HPLC/MSD		Mastoeidis		3			Tyrosol, vanillic acid, luteolin, apigenin			[86]	
^1^H-NMR		13 cultivars *n* **		158			Oleocanthal, oleacein			[40]	
LC-MS		Koroneiki Lianolia		20 20			Hydroxytyrosol, tyrosol, oleacein aglycon, aldehydic form of oleuropein aglycon, oleocanthal aglycon, aldehydic form of ligstroside aglycon, *p*-coumaric acid, ferulic acid, vanillic acid, 1-acetoxypinoresinol, apigenin, luteolin			[87]	
HPLC-GCMS		Lianolia		Not provided			Hydroxytyrosol, tyrosol, and derivatives			[88]	
HPLC		Koroneiki		20			Total hydroxytyrosol and tyrosol			[11]	
HPLC-Orbitrap-HRMS/MS		Koroneiki		Not provided			Identified 25 compounds, Quantitated: total hydroxytyrosol, total tyrosol, oleuropein aldehydic form, oleuropein aglycon, oleuropein and ligstroside aldehydic form, oleocanthal, oleacein			[67]	
HPLC		Throumbolia, Koroneiki 3 ripening stages		6			Total hydroxytyrosol and tyrosol, oleuropein aglycon, dialdehydic form of elenolic acid linked to hydroxytyrosol, dialdehydic form of elenolic acid linked to tyrosol			[31]	
GC-MS, TMS derivatives		Koroneiki		1			Free hydroxytyrosol and tyrosol, *p*-coumaric acid, vanillin, vanillic acid, *p*-hydroxybenzoic acid, ferulic acid, *p*-hydroxyphenylacetic acid, homovannilic alcohol, kaempferol			[89]	
GC-MS, TMS derivatives		Koroneiki		1			Free hydroxytyrosol and tyrosol, caffeic acid, *p*-coumaric acid, vanillin, vanillic acid, *p*-hydroxybenzoic acid, ferulic acid, *p*-hydroxyphenylacetic acid, syringic acid, cinnamic acid, homovannilic alcohol, protocatechuic acid, kaempferol			[90]	
GC-MS, TMS derivatives		Koroneiki 2 crop years organic *vs.* conventional		32			Free hydroxytyrosol and tyrosol, caffeic acid, *p*-coumaric acid, vanillic acid, ferulic acid, *p*-hydroxybenzoic acid, syringic acid, cinnamic acid, homovannilic alcohol, protocatechuic acid			[34]	
GC-MS, TMS derivatives		Koroneiki 3 ripening stages		3			Free hydroxytyrosol and tyrosol, caffeic acid, *p*-coumaric acid, vanillic acid, *p*-hydroxybenzoic acid, ferulic acid, cinnamic acid, homovannilic alcohol, kaempferol, naringenin, genistein			[35]	

NS: not specified; total hydroxytyrosol and total tyrosol: the sum of free and esterified forms of both phenyl alcohols; *: Mastoeidis cultivar is also referred as “Athinolia” or “Tsounati”; **: Adramytini, Agouromanaki, Athinolia, Chalkidiki, Conservolia, Kolovi, Koroneiki, Koutsourolia, Lianolia, Manaki, Megaritiki, Throuba, Sylvestris; TMS: trimethylsilyl.

**Table 3:** Tyrosol and hydroxytyrosol (mg/kg) in Greek monovarietal EVOO obtained during five harvesting periods (2002–2006 & 2007–2008) [28].

Area	Cultivar	Total Hydroxytyrosol	Total Tyrosol	Free Hydroxytyrosol	Free Tyrosol	*N*
Crete	Koroneiki	8.6–330	8.9–54.5	nd-6.3	nd-5.7	95
Peloponnisos	Koroneiki	3.4–132	9.1–40.3	nd-8.4	0.2–10.7	47
Zakynthos Island	Koroneiki	13.1–83.0	23.8–81.2	0.1–2.9	0.3–7.1	21
Crete	Mastoeidis	14.7–432	16.1–136	0.1–25.4	0.7–46.6	19
Peloponnisos	Mastoeidis	13.7–131	27.1–131	0.4–10.2	nd-8.4	21
Crete	Throumbolia	52.1–201	40.3–87.8	5.0–19.3	nd-6.6	5
Lesvos Island	Adramytini	7.1–121	7.7–72.9	nd-12.5	0.7–23.4	13

nd: not detected; typical values for individual phenols content of olive oils are also available in the Web [91].

Currently, good practices from farm to olive mill and then throughout the distribution line, along with awareness of virgin olive oil consumers about the health benefits of minor constituents of this expensive oil had an impact on the quality of the end product. A shift of the type of container in retail market from transparent glass or polymer bottle to dark glass, tin to UV lined polymers has an impact on upholding high levels of tocopherols throughout the shelf life of an extra VOO. Among the Greek cultivars for oil production Koroneiki cv, native to South western Peloponnisos, prevails. The oil of this cultivar is acknowledged for its fruity and pungent flavor as well as its pleasant green color. No studies were found on the potential of this cultivar regarding tocopherol content. However the finding reported by Dabbou *et al.* (2011) for experimental oils from Koroneiki cv grown in Tunisia is very promising and needs further investigation [92]. The oils that were extracted using an Abencor system (MC2 Ingenierias y Sistemas, Sevilla, Spain) were found to contain from 409 to 638 mg α-T/kg. Cold pressed or cold extracted VOO claim applies only for virgin or extra virgin olive oils obtained at a temperature below 27 °C by pressing of the olive paste by a traditional extraction system using hydraulic presses, percolation or centrifugation of the olive paste [93]. These oils that contain higher amounts of minor constituents such as the polar phenol content, responsible for the bitter/pungent hues in taste, should also contain high levels of tocopherols. The latter depends heavily on the cultivar potential. Chalkidiki Chondrolia cv that ripens green seems to contain much less tocopherols in comparison to Koroneiki cv oils but is richer in polar phenolic compounds [94]. Altitude may be another factor that has to be considered, as VOOs from mountainous areas are now recognised as a particular category. A slight decrease in tocopherol content of Mastoides cv. grown in Chania region has been reported from sea level up to 800 m [95].

## 4. Squalene

Squalene (2,6,10,15,19,23-hexamethyl-2,6,10,14,18,22-tetracosahexane), found in human, animal, plant and microbe cells as a precursor of sterols and of many other bioactive terpenoids, is the major component of VOO non-saponifiable fraction. The physicochemical properties, biosynthesis, natural occurrence, chemical synthesis and industrial sources of squalene have been recently reviewed [96].

Its 10 fold higher intake in the Mediterranean countries than that in northern European countries or the United States has been related to the consumption of olive oil that might explain the low incidence of certain forms of cancer in Mediterranean populations [97,98]. Bioactivity studies so far indicated functional properties of squalene, among which *in vivo* antioxidant activity, and attracted the interest in search of new sources including biotechnology [99]. Absorption of squalene is high, 60% to 85%. It is transported to serum in association with low-density lipoproteins and distributed to the adipose tissue, skin, pancreas and liver [100,101]. Dietary intake of squalene was not connected with increase in the levels of cholesterol in plasma [102]. Squalene content in plant sources varies considerably (0–12 g/kg). Among edible sources VOO prevails (~6.1 g/kg oil) followed by that in pumpkin seed and rice bran oils (~3.1 and 1.5 g/kg, respectively). Crude palm oil contains also squalene (mean value ~1.0 g/kg). A contribution of analytical protocols used to variation in literature values should be stressed [73]. The genetic factor may influence these levels but existing studies are not conclusive as in the case of polar phenolic compounds [103,104,105]. Processing such as extraction and mainly refining reduce the squalene content [106,107,108]. Bleaching gives rise to the formation of isomers ~3% of C_30_H_50_ content can be isomerized [109]. Its level in deodorized OO is expected to be 10 times lower than that in an EVOO. Thus, deodorization distillates are one of the by-products used to recover squalene together with tocopherols and sterols. The contribution of squalene to olive oil stability under light exposure or in the dark is not fully investigated and the results are non conclusive [99,105,110,111]. It is more possible that its activity is related to competitive phenomena [70], and not to a radical scavenging mechanism as it is repeated sometimes in literature without justification. Experiments about the contribution of squalene to the radical scavenging activity of olive oil using the DPPH radical proved that when squalene, α-tocopherol or caffeic acid were added to olive oil substrate devoid of polar phenols (DPP) the addition of the former did not exert any increase in the radical activity of the substrate (Table 4) (Naziri and Tsimidou, 2013) [112].

Naziri and Tsimidou [113] characterized the oxidation products of squalene under various conditions and examined their prooxidant activity in an olive oil substrate showing that they actively participate in propagation reactions. Loss upon heating seems to be related to conditions and the presence of food that is cooked [114,115]. Overall, squalene is considered as a stable molecule under autoxidation conditions.

Squalene levels in samples from retail Greek market and from the Greek cultivars Mavrolia and Koroneiki were within the levels reported in the literature for high quality VOO (2000–5858 mg/kg) [35,110,111,116,117,118]. A decreasing trend with ripening has been observed in Koroneiki VOO [34,35]. No systematic studies were found in literature. For the non typical production year 2002–2003 in Greece that produced lower quality oils, VOOs (*n* = 28) from Koroneiki cv originating from the two major oil producing areas in Greece, Crete and Peloponnisos, showed average levels of total polar phenolic compounds, α-tocopherol, and squalene 150 mg CAE/kg, 182 mg/kg and 3500 mg/kg (Tsotsou and Tsimidou, 2004) [119]. This finding indicates dependence from climatic conditions.

**Table 4:** Radical scavenging activity (RSA) of an olive oil devoid of polar phenolic compounds (DPP) after the addition of squalene, α-tocopherol or caffeic acid at realistic levels (Naziri and Tsimidou) [112].

Sample	% RSA (Mean ± SD, *n* = 3)
DPP *	62.7 ± 0.1
(a) after SQ addition	
DPP + 5000 mg/kg	63.1 ± 0.3
DPP + 10,000 mg/kg	62.9 ± 0.6
DPP + 15,000 mg/kg	63.9 ± 0.3
(b) after α-tocopherol addition	
DPP + 150 mg/kg	76.8 ± 0.3
DPP + 350 mg/kg	94.7 ± 0.8
DPP + 700 mg/kg	95.7 ± 1.2
(c) after caffeic acid addition	
DPP + 50 mg/kg	82.9 ± 0.3
DPP + 100 mg/kg	92.9 ± 0.9
DPP + 150 mg/kg	97.9 ± 0.9

* Composition of DPP: total polar phenolic compounds: not detectable using the Folin-Ciocalteu method; squalene: 4518 ± 193 mg/kg; α-tocopherol: 175 ± 10.8 mg/kg.

## 5. Triterpenic Acids

Triterpenic compounds are common constituents of plants, occurring in the form of free acids or aglycones of triterpenoid saponins. They are relatively non-toxic and possess pharmacological properties exerting anti-inflammatory, hepatoprotective, antitumor, antiviral, anti-HIV, antimicrobial, antifungal, antidiabetic, gastroprotective and antihyperlipidemic action in experimental studies and animal models [120]. In the literature there are scarce data for terpenic acids in olive oil. The hydroxy pentacyclic triterpenic acids (HPTA) oleanolic acid (3β-hydroxyolean-12-en-28-oic acid), ursolic acid (3β-hydroxyurs-12-en-28-oic acid), maslinic acid (2α,3β-dihydroxyolean-12-en-28-oic acid) and betulinic acid (3β-hydroxylup-20(29)-en-28-oic acid) occur in small amounts in olive oils [3]. As triterpenes are concentrated mainly in the skin of fruits, their concentrations in olive oils are several times lower than in pomace olive oil. In a comprehensive study employing olive oils from Italian, Moroccan, Spanish and Tunisian cultivars, the reported HPTA content ranged between 38 and 145 mg/kg for EVOO, 312 and 583 mg/kg for VOO, 2385 and 10,088 mg/kg for crude pomace oil [121], with oleanolic acid representing 45%–65% of total HPTA in EVOO and VOO and 82%–91% in pomace oil; it was also documented that refining caused significant HPTA losses. In a study of the terpenoids content in VOO obtained from 40 olive cultivars grown in the World Olive Germoplasm Bank Collection of Cordoba, Spain, including two Greek varieties, Valanolia and Megaritiki [122], the sum of HPTA ranged between 8.90 and 112.36 mg/kg (mean 34.76 mg/kg); oleanolic and maslinic acids ranged between 3.39 and 78.83, 3.93 and 49.81 mg/kg, respectively, and ursolic acid was present in trace amounts, lower than 4 mg/kg. The HPTA contents of the Greek varieties Valanolia and Megaritiki were 41.19 and 22.22 mg/kg with oleanolic acid comprising the 52% and 60% of total HPTA, respectively [122]. The HPTA content of VOO of the Koroneiki cultivar from Southern Peloponissos was not affected by the type of cultivation–organic *vs.* conventional, in a study covering 2 harvesting periods [34]. Laboratory-scale experiments with Spanish olive varieties indicated that VOO triterpenic composition can be improved by regulating the preparation conditions of olive paste [123]. A recent study on the effect of olives’ maturation on several phytochemicals in organic Koroneiki EVOO from Messenia, Peloponnisos, revealed that ripening caused a decrement of HPTA from 68.56 mg/kg in oils obtained from olives at early maturity to 13.0 mg/kg in oils obtained from mature olives, with oleanolic acid comprising the 79% and 70% of total HPTA, respectively [35]. The decrement of HPTA with olive maturation has been also reported for oils of the Picual variety in Spain [121]. In a recent study on the fate of selected secondary metabolites during Koroneiki oil production, maslinic acid increased from 1252.9 mg/kg in the drupes to 3123.2 mg/kg in the paste, and decreased significantly in the first and second oil reaching 29.5 and 20.9 mg/kg, respectively [67]. The extraction technology did not affect significantly the levels of oleanolic, ursolic and maslinic acids in cold pressed EVOO produced with 2-phase and 3-phase decanters from mature olives of the Koroneiki cultivar, collected from the same olive field in Messenia [124]. In this case the HPTA content of 2-phase and 3-phase EVOO was 15.6 and 14.8 mg/kg with oleanolic acid representing the 69% and 71% respectively. Up to now no reports exist on the effect of storage on the HPTA content of VOO.

## 6. Storage and Use

The extent of quality deterioration of VOO depends on the storage conditions—temperature, light, presence of oxygen in headspace, headspace volume—and on the package material. The changes expected are mainly caused by hydrolytic and oxidative phenomena. During storage, VOO antioxidants, being more oxidisable, spare the shelf life of the polyunsaturated fatty acids. Upon storage the complex forms of hydroxytyrosol and tyrosol are reduced and the levels of the latter increase. Free forms are oxidised easily so that upon oil ageing a reduction in TPC is expected. The changes in the content and type of polar phenolic content affect the sensory characteristics of the oil. Less bitter and pungent oils are obtained even after six months of storage. In a series of studies on the stability of Greek VOOs in the dark or under light exposure Psomiadou and Tsimidou [70,110,111] followed changes in the content within one class or among classes of antioxidants together with the changes in the stability of the oil. Their overall findings can be summarized as follows: (a) oxygen availability is crucial in all cases; (b) light exposure is destructive for the VOO shelf life due to the significant levels of pheophytin a present, which acts as a strong prooxidant; (c) The presence of chain breaking antioxidants and radical scavengers, cover up and diminish the contribution of squalene to VOO stability; (d) under autoxidation conditions, the loss of α-tocopherol and carotenoids was comparable with that of polar phenol content; (e) loss of carotenoids in the dark was significant due to the chemical mechanism involved, whereas under light exposure carotenoids act as filters quenching light energy by physical mechanism. In studies with Koroneiki VOO conducted in Epirus (NW Greece) and Crete, storage temperature, light and to a lesser extent headspace oxygen were shown to cause similar effects [125,126]. Regarding the packaging material, it was shown that the most appropriate material for VOO packaging is glass followed by polyethylene terephthalate (PET), while the oil should preferably be kept in dark colored containers at the dark, avoiding temperatures higher than 22 °C [125]. In the same study it was concluded that containers with high oxygen transmission ratios such as polypropylene and polyethylene are not suitable for the packaging of olive oil [125]. The presence of oxygen in the headspace of filtered and unfiltered Koroneiki VOO from Crete stored outside was shown to be an important factor in controlling VOO quality during storage as after 15 months of outside storage the filtered oils with N_2_ in headspace presented the higher retention of TPC (around 70%), while the unfiltered samples under air had the worse retention [126]. Regarding individual phenols, the major secoiridoid derivatives, namely the dialdehydic form of elenolic acid linked to hydroxytyrosol and the oleuropein aglycon decreased, whereas the simple phenolic compounds tyrosol and hydroxytyrosol increased gradually during storage, in agreement with previous reports [26,127]. Loss of stability is faster in filtered oils than in the unfiltered (veiled) ones [26]. Home practices accelerate the above mentioned changes monitored under controlled conditions. Consumers should buy this precious oil at quantities that correspond to the needs of the number of the persons of a family. Storage in the dark at ambient temperature is essential. Avoidance conduct with direct light is a prerequisite. Extra virgin olive oil is for the majority of consumers worldwide a salad oil. For traditional consumers is the basic or even the only fat used in culinary practices.

## 7. Conclusions

Compositional data for the profiles and levels of polar phenolic compounds, tocopherols, squalene and triterpenic acids revealed that the composition of endogenous antioxidants of Greek virgin olive oils deserves further and systematic investigation. The published data of similar investigations will strengthen the competitiveness and reputation of this precious national product among world virgin olive oils. The major variety in Greece for VOO production is the Koroneiki cv. This produces oils of medium to high TPP content, rich in bound forms of hydroxytyrosol and tyrosol. Published data for Greek VOOs are not as extensive as those available for Spanish and Italian ones. For this reason, it is important to construct a national databank with all published and unpublished data produced by academia, institutes and the relevant Greek authorities for the composition and levels of these bioactive compounds. Such a databank should be updated regularly and can serve as a reference in all future efforts for promotion of Greek virgin olive oil or discussions about various types of claims (e.g., health claims for polar phenolic compounds) at international fora.
